# 

**Published:** 1888-12

**Authors:** 


					﻿Entered at the Post Office at Austin, Texas, as Second Class Mall Matter.
Vol. IV.
DECEMBER, 1888.
No. 6
DANIEL'S
MEDICAL
JOURNAL
F. E. DANNIEL, M. D., Editor and Publisher, AUSTIN, TEXAS.
Eugene Von Boeckmann, Steam Book and Job Printer, Austin, Texas.
				

